# Use of the slide positivity rate to estimate changes in malaria incidence in a cohort of Ugandan children

**DOI:** 10.1186/1475-2875-8-213

**Published:** 2009-09-15

**Authors:** Trevor P Jensen, Hasifa Bukirwa, Denise Njama-Meya, Damon Francis, Moses R Kamya, Philip J Rosenthal, Grant Dorsey

**Affiliations:** 1 Department of Medicine, University of California, San Francisco, USA; 2 Uganda Malaria Surveillance Project, Kampala, Uganda; 3 Makerere University Medical School, Kampala, Uganda; 4 San Francisco General Hospital, 1001 Potrero Avenue, Building 30, Room 408 San Francisco, CA 94110, USA

## Abstract

**Background:**

As malaria control efforts intensify, it is critical to monitor trends in disease burden and measure the impact of interventions. A key surveillance indicator is the incidence of malaria. Yet measurement of incidence is challenging. The slide positivity rate (SPR) has been used as a surrogate measure of malaria incidence, but limited data exist on the relationship between SPR and the incidence of malaria.

**Methods:**

A cohort of 690 children aged 1-10 years at enrollment were followed for all their health care needs over a four-year period in Kampala, Uganda. All children with fever underwent laboratory testing, allowing us to measure the incidence of malaria and non-malaria fevers. A formula was derived to estimate relative changes in the incidence of malaria (rΔIm) based on changes in the SPR and the assumption that the incidence of non-malaria fevers was consistent over time. Observed and estimated values of rΔIm were compared over two, six, and 12 month time intervals after restricting the analysis to children contributing observation time between the ages of 4-10 years to control for aging of the cohort.

**Results:**

Over the four-year observation period the incidence of malaria declined significantly from 0.93 episodes per person-year in 2005 to 0.39 episodes per person-year in 2008 (p < 0.0001) and the incidence of non-malaria fevers declined significantly from 2.31 episodes per person-year in 2005 to 1.31 episodes per person-year in 2008 (p < 0.0001). Younger age was associated with a significantly greater incidence of malaria and the incidence of malaria was significantly higher during seasonal peaks occurring each January-February and May-June. Changes in SPR produced reasonably accurate estimates of rΔIm over all time intervals. The average absolute difference in observed and estimated values of rΔIm was lower for six-month intervals (0.13) than it was for two-month (0.21) or 12 month intervals (0.21).

**Conclusion:**

Changes in SPR provided a useful estimate of changes in the incidence of malaria in a well defined cohort; however, a gradual decline in the incidence of non-malaria fevers introduced some bias in these estimates.

## Background

Malaria remains one of the most important global health challenges, with an estimated three billion people at risk of infection, leading to approximately 250 million cases and one million deaths each year [1]. Efforts to reduce the burden of malaria have intensified recently through the use of effective tools for malaria control, notably long-lasting insecticide-treated nets (LLITN) and artemisinin-based combination therapy (ACT), backed by indoor residual spraying (IRS) of insecticides. These efforts have been made possible by improved policy recommendations and increased support from governments, international organizations, and donors. These expanding resources have come with ambitious targets for control and expectations of significant reductions in disease burden. As an example, in 2005 the World Health Assembly set a target of reducing malaria cases and deaths per capita by 50% between 2000 and 2010, and by 75% between 2005 and 2015 [2].

As malaria control efforts intensify, it is critical to monitor disease burden and trends, and to measure the impact of interventions [3]. The incidence of malaria, defined as the number of confirmed malaria cases per person-time, is one of the key indicators for monitoring and evaluation [1]. The general working definition of a case of malaria is considered to be "fever with malarial parasitaemia", which normally defines all patients that require anti-malarial treatment [1]. The most accurate method of estimating malaria incidence involves longitudinal studies in defined populations, where all cases of suspected malaria (i.e. those with fever) are captured and undergo a diagnostic test that is highly sensitive and specific. However, such methods require considerable resources and are rarely undertaken as part of routine malaria surveillance programmes. Malaria incidence is typically estimated based on the number of reported cases of malaria captured through a country's health management information system (HMIS). However, there is often a significant lag time before HMIS data become available. In addition, incomplete reporting, lack of a clear denominator, and the absence of laboratory confirmation limit the accuracy of these data [4], particularly in African countries where the vast majority of reported cases of malaria are not laboratory confirmed [3]. Although statistical adjustments can improve the accuracy of estimates of malaria incidence based on HMIS data, temporal variations in reporting, varied utilization of health care services, and limitations on the accuracy of diagnostic testing can lead to considerable bias and confound attempts to define the relationship between malaria incidence and control interventions.

The slide positivity rate (SPR), defined as the number of laboratory-confirmed malaria cases per 100 suspected cases examined, provides an alternative method for estimating temporal changes in malaria incidence. Compared to clinical definitions of malaria, the SPR gains accuracy in considering only laboratory confirmed cases of malaria, and it can provide a rapid and inexpensive means of assessing the burden of malaria in a population utilizing health care facilities. The SPR has been used in cross-sectional studies to define the level of malaria endemicity [5,6]. Decreases in the SPR have been cited as evidence for successful malaria control interventions in Africa [7,8], yet there has been little work describing the quantitative relationship between temporal changes in SPR and changes in malaria incidence using empiric data.

To better characterize the quantitative relationship between SPR and the incidence of malaria we compared changes in these metrics over a 4 year period within a cohort of children in Kampala. This cohort was characterized by a highly incentivized study protocol designed to maximize the capture of all illnesses, uniform diagnostic testing for all episodes of fever, and a highly accurate diagnostic testing system. This study allowed us to directly compare observed changes in the incidence of malaria with changes in incidence of malaria estimated from the SPR.

## Methods

### Study site and recruitment of study participants

Subjects were recruited from the Mulago III parish of Kampala, Uganda, a densely populated urban slum approximately 1 square kilometer in area. Malaria in Kampala is meso-endemic, with two peaks in incidence following the rainy seasons of December-January and April-May [9]. Prior to the start of the study, a census survey was conducted within the parish to provide a sampling frame for study recruitment, as previously described [10]. Between November 2004 and April 2005, 601 children from households randomly selected from our census survey were enrolled in a prospective cohort study if they fulfilled all of the following eligibility criteria: 1) age 1 to 10 years; 2) agreement to come to the study clinic for any febrile episode or illness; 3) agreement to avoid medications administered outside the study; 4) agreement to remain in Kampala during the study period; 5) no known side effects to the study medications; 6) weight > 10 kg; 7) absence of severe malnutrition or known serious chronic disease; 8) absence of life threatening screening laboratory results; and 9) willingness of parent or guardian to provide written informed consent. An additional 89 children were enrolled February - May 2007 using the same eligibility criteria. These children were living in the same households as previously enrolled children but either too young or not yet born at the time of the initial recruitment period.

### Follow-up of study participants

Details of the study follow-up have been previously described [11] and are summarized here. Participants were asked to come to a designated study clinic, open 7 days a week, for all healthcare needs. Subjects who presented to the clinic with fever (tympanic temperature ≥ 38.0°C) or reported history of fever symptoms in the previous 24 hours had blood obtained by fingerprick for a thick blood smear. If the thick blood smear was positive, the participant was diagnosed with and treated for malaria regardless of the parasite density. The first time a subject was diagnosed with uncomplicated malaria, he/she was randomized to receive one of the following anti-malarial combination therapies: amodiaquine + sulphadoxine-pyrimethamine (AQ + SP), amodiaquine + artesunate (AQ + AS), or artemether-lumefantrine (AL). For any subsequent episode of uncomplicated malaria during the remainder of the study, subjects received the same therapy to which they were randomized. Subjects who had recurrence of symptomatic malaria within 14 days were given quinine. Participants with severe malaria or danger signs were also treated with quinine. If, upon any presentation, the thick blood smear was negative, the participant was not given anti-malarial therapy and was managed at the discretion of the study physicians. The diagnosis and management of malaria was based on initial readings of blood smears. Final microscopy results were based on a rigorous quality control system which included re-reading all blood smears by a second microscopist and resolution of any discrepancies by a third microscopist. Of note, no patients diagnosed with uncomplicated malaria based on the initial smear reading were deemed to have a negative smear following the quality control readings.

Data on ITN use was collected at enrollment, although no intervention was made at that time. All participants were given a LLITN between May and June of 2006. In March 2007, the AQ + SP treatment arm was dropped due to poor efficacy. In January 2008 the AQ + AS treatment arm was dropped because the AS component of this regimen was no longer available in Uganda. Study participants were prematurely withdrawn from the study if they met any of the following criteria: 1) movement out of the study area, 2) inability to locate study participants for > 60 consecutive days, 3) withdrawal of informed consent, and 4) inability to comply with the study schedule and procedures. Observation for new episodes of malaria was terminated on December 15^th^, 2008, and the study was completed on January 31, 2009.

### Statistical analysis

Data was double-entered in Access (Microsoft Corporation, Redmond, WA) and analyzed using STATA Version 10 (College Station, TX) and Microsoft Excel (Microsoft Corp. Redmond, Wa). To control for changes in the age of the cohort over time, data were only included for children who were 4-10 years of age between January 2005 and December 2008. The incidence of malaria (Im) was defined as the number of treatments for malaria divided by the person-time of observation in years. SPR was defined as the number of treatments for malaria (all based on a positive blood smear) divided by the total number of blood smears done among study participants who presented with an episode of fever. The observed relative change in the incidence of malaria (rΔIm) was defined as Im_*i*+1_/Im_*i*_, where *i *represents the baseline time interval and *i+1 *represents the interval immediately following. To estimate rΔIm using SPR, the following assumptions were made: 1) all fevers in the study population were captured, 2) all study participants who presented with fever underwent microscopy, and 3) the sensitivity and specificity of microscopy was 100%. Given these assumptions, during any time period SPR can be defined by the following equation:



where Inm represents the incidence of fever not due to malaria. If Inm does not change from one time interval to the next, rΔIm can be estimated from the SPR by the following formula:



where *i *represents the baseline time interval and *i+1 *represents the interval immediately following. The observed and estimated rΔIm were compared across two month, six month, and 12 month time intervals. Relative changes in incidence were always made in reference to consecutive intervals of time such that over the 4 year observation periods there were 23, 7, and 3 estimates of rΔIm across two, six, and 12 month intervals, respectively. Agreement between the observed and estimated values of rΔIm for these intervals was assessed using three methods; directionality (relative increase or decrease in the incidence of malaria), overestimation versus underestimation, and average absolute difference in rΔIm.

Continuous variables were compared using the t-test for two-group comparisons and ANOVA for comparisons of three or more groups. Comparisons of the incidence of malaria and non-malaria fever were made using a negative binomial regression model with exposure reflected by the observation time. A p-value < 0.05 was considered statistically significant.

## Results

### Description of cohort

The characteristics of the study cohort stratified by the year of observation are summarized in Table 1. Of the 690 children enrolled in the cohort, 574 were observed between the ages of 4-10 years contributing a total of 1,266 person-years of follow-up with a median follow-up time per child of 2.01 years (IQR 1.03-3.33 years). The mean age of the children followed in 2005 was significantly lower than that of children observed during the subsequent three years (6.80 vs. 7.16 years, p = 0.0006), but mean age was not significantly different between the final three years of follow-up (p = 0.82). The proportion of observation days with ITN coverage was low (5.4%) during the first year of the study but increased to almost 100% in May of 2006 when all of the study participants were given LLITNs. Changes in the proportion of different anti-malarial treatment regimens given reflected changes in treatment efficacy and the study protocol. AQ + SP made up of the largest proportion of treatments in 2005-06 due to its lower efficacy (and, therefore, increased need for retreatment of failures) [11] and this regimen was dropped in 2007. The AQ + AS treatment arm was dropped in early 2008, making AL the predominant treatment used in the final year of observation. The use of quinine for complicated malaria and treatment failures made up only a small proportion (2.5%) of the total treatments.

**Table 1:** Characteristics of cohort stratified by year of observation

**Characteristics**	**Year of observation**			
	
	**2005**	**2006**	**2007**	**2008**
Number of children*	433	425	396	319
Person-years of follow-up	349	348	312	258
Mean age in years (SD)	6.80 (1.81)	7.11 (1.93)	7.19 (1.93)	7.19 (1.84)
ITN coverage^†^	5.4%	64.4%	99.8%	100%
Total blood smears performed	1133	937	650	438
Positive blood smears	326	302	196	101
Malaria treatment given^‡^				
AQ + SP	123	123	24	0
AQ + AS	111	79	71	8
AL	83	94	94	92
Quinine	9	6	7	1
Slide positivity rate (SPR)	28.8%	32.2%	30.2%	23.1%
Incidence of malaria^£^	0.93	0.87	0.63	0.39
Incidence of non-malaria fever^£^	2.31	1.83	1.46	1.31

*Number of children contributing at least one day of follow up to yearly analysis

^†^Proportion of observation days with ITN coverage

^‡^AQ = amodiaquine; SP = sulfadoxine-pyrimethamine; AS = artesunate; AL = artemether-lumefantrine

^£^per person-year

### Incidence of malaria and non-malaria fever

Over the four-year observation period there were a total of 925 episodes of malaria and 2,233 episodes of non-malaria fever, resulting in an incidence of malaria of 0.73 per person-year, and an incidence of non-malaria fever of 1.76 per person-year. Observed data for the incidence of malaria and non-malaria fever over two-month time intervals are presented in Table 2. Both the incidence of malaria and the incidence of non-malaria fever were significantly associated with age and time of year (Table 3). Children 4-5 years of age had a similar incidence of malaria compared to those 6-7 years of age (0.91 vs. 0.72 episodes per person-year, p = 0.22), but a significantly higher incidence of malaria compared to those 8-10 years of age (0.91 vs. 0.58 episodes per person-year, p = 0.001). Children 4-5 years of age had a significantly higher incidence of non-malaria fever compared to older children (2.18 vs. 1.58 episodes per person-year, p < 0.0001) but the incidence of non-malaria fever was similar for those 6-7 years of age compared to those 8-10 years of age (1.66 vs. 1.50 episodes per person-year, p = 0.13). The incidence of malaria was significantly higher during the months of January-February and May-June following the two rainy seasons compared to the rest of the year (1.18 vs. 0.51 episodes per person-year, p < 0.0001). The incidence of non-malaria fever had a single peak during the months May-June that was significantly higher compared to the rest of the year (2.52 vs. 1.61 episodes per person-year, p < 0.0001). Over the four-year observation period the incidence of malaria declined significantly from 0.93 episodes per person-year in 2005 to 0.39 episodes per person-year in 2008 (p < 0.0001) (Table 1). Over the four-year observation period the incidence of non-malaria fevers also declined significantly from 2.31 episodes per person-year in 2005 to 1.31 episodes per person-year in 2008 (p < 0.0001) (Table 1). The majority of non-malaria fever cases (80%) were due to one of four diagnoses; common cold, pharyngitis, upper respiratory tract infection, and fever not otherwise specified.

**Table 2:** Smear positivity rate and incidence of malaria and non-malaria fever over time

**Year**	**Months**	Total BS*	Positive BS*	**Person-years**	**SPR**	**Incidence of malaria^†^**	**Incidence of non-malaria fevers^†^**
2005	Jan-Feb	132	67	34.5	50.80%	1.94	1.88
	Mar-Apr	179	51	55.7	28.50%	0.92	2.3
	May-Jun	304	81	62.9	26.60%	1.29	3.54
	Jul-Aug	208	50	65.5	24.00%	0.76	2.41
	Sept-Oct	164	29	65.9	17.70%	0.44	2.05
	Nov-Dec	146	48	64.7	32.90%	0.74	1.52

2006	Jan-Feb	154	63	58.8	40.90%	1.07	1.55
	Mar-Apr	165	55	58	33.30%	0.95	1.9
	May-Jun	260	95	57.2	36.50%	1.66	2.88
	Jul-Aug	133	26	57.9	19.50%	0.45	1.85
	Sept-Oct	104	19	57.9	18.30%	0.33	1.47
	Nov-Dec	121	44	57.7	36.40%	0.76	1.33

2007	Jan-Feb	156	89	52.5	57.10%	1.7	1.28
	Mar-Apr	100	25	53.2	25.00%	0.47	1.41
	May-Jun	127	32	52.8	25.20%	0.61	1.8
	Jul-Aug	101	13	53	12.90%	0.25	1.66
	Sept-Oct	86	16	51.1	18.60%	0.31	1.37
	Nov-Dec	80	21	49	26.30%	0.43	1.2

2008	Jan-Feb	74	27	46.4	36.50%	0.58	1.01
	Mar-Apr	78	12	46.3	15.40%	0.26	1.43
	May-Jun	100	32	45.5	32.00%	0.7	1.49
	Jul-Aug	73	11	45.3	15.10%	0.24	1.37
	Sept-Oct	62	8	43.4	12.90%	0.18	1.24
	Nov-Dec	51	11	31	21.60%	0.35	1.29

*blood smear

^†^per person-year

**Table 3:** Associations between age and seasonality the incidence of malaria and non-malaria fever.

**Incidence of malaria**						
**Category**	**Group**	**Episodes**	**Person years**	**Incidence**	IRR* **(95% CI)**	**p-value**

Age	4-5 years	358	394	0.91	1.0 (reference)	-
	6-7 years	303	418	0.72	0.85 (0.66-1.10)	0.22
	8-10 years	264	454	0.58	0.64 (0.50-0.84)	0.001

Season	Mar-Apr + Jul-Dec	439	856	0.51	1.0 (reference)	-
	Jan-Feb + May-Jun	486	411	1.18	2.52	< 0.001

**Non-malaria fever**						

Age	4-5 years	857	394	2.18	1.0 (reference)	-
	6-7 years	693	418	1.66	0.75 (0.64-0.87)	< 0.001
	8-10 years	683	454	1.5	0.66 (0.57-0.77)	< 0.001

Season	Rest of the year	1682	1048	1.61	1.0 (reference)	-
	May-Jun	551	219	2.52	1.57 (1.40-1.77)	< 0.001

*Incidence rate ratio estimated using a negative binomial regression model

### Comparison of observed and estimated changes in the incidence of malaria

**Figure 1:**
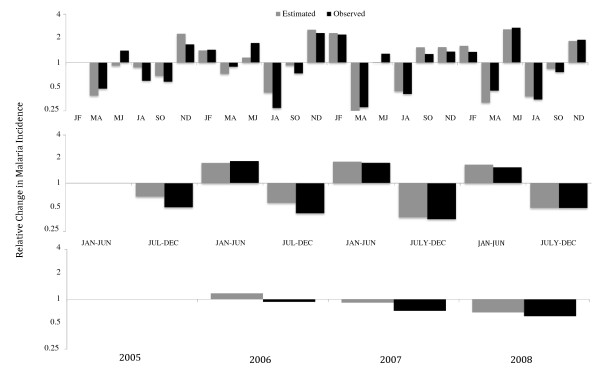
**Estimated vs. Observed Change in Malaria Incidence**. Estimated versus observed relative change in the incidence of malaria (incidence of malaria in the current time interval/incidence of malaria in the prior time interval) over two, six, and 12 month intervals between January 2005 and December 2008. Y-axis expressed in terms of the incidence rate ratio (i.e. 2 equals a 2-fold increase in the incidence of malaria and 0.5 equals a 2-fold decrease in the incidence of malaria) and X-axis expressed in terms of calendar time.

Relative changes in the incidence of malaria (rΔIm) using observed data and that estimated from the SPR were compared over two, six and 12 month intervals (Figure 1). Using two-month time intervals the relative change in the incidence of malaria ranged from 0.27-2.71 for the observed values and 0.25-2.59 for the estimated values. Using six-month time intervals the relative change in the incidence of malaria ranged from 0.35-1.80 for the observed values and 0.37-1.86 for the estimated values. Using 12 month time intervals the relative change in the incidence of malaria ranged from 0.62-0.93 for the observed values and 0.69-1.18 for the estimated values. The observed and estimated rΔIm had the same directionality (relative increase or decrease in the incidence of malaria) in 22 of 23 (96%) two-month intervals, seven of seven (100%) six-month intervals, and two of three (67%) 12 month intervals. The estimated rΔIm was higher than the observed rΔIm in 13 of 23 (57%) two-month intervals, six of seven (86%) six-month intervals, and three of three (100%) 12 month intervals. The average absolute difference in observed and estimated values of rΔIm was lower for six-month intervals (0.13) than it was for either two-month (0.21) or 12 month intervals (0.21).

## Discussion

As efforts to reduce the burden of malaria increase, there is a vital need to accurately measure the impact of control interventions. The vast majority of malaria control interventions are not implemented as controlled experiments, therefore it is important to develop standardized metrics and methods for estimating their impact through routine surveillance. One of the most important metrics for malaria monitoring and evaluation is the incidence of disease (i.e. the number of cases of malaria per person-time). However, direct measures of malaria incidence are expensive and logistically challenging in the context of routine surveillance programmes, especially within Africa. The SPR has been used as a practical and relatively inexpensive alternative for tracking the burden of malaria over time. Indeed, the use of the SPR is advocated by the World Health Organization [1] and SPR measures have been used as evidence of both temporal increases [12] and decreases in the burden of malaria [7]. For example, routine surveillance data from four sites in The Gambia documented a decline in the SPR from 11-37% in 2003 to 2-18% in 2007. This substantial reduction in SPR was temporally linked to a more than three-fold increase in ITN coverage and a change in anti-malarial treatment policy [7]. Nevertheless, SPR remains a surrogate measure of the burden of malaria and a better understanding of its relationship to malaria incidence is needed.

Based on the assumptions that there is no differential bias in the subgroup of patients with suspected malaria who undergo laboratory testing within a target population, and that the incidence of non-malaria fevers does not change over time, we show that there is a direct mathematical relationship between the relative change in the incidence of malaria (rΔIm) and the relative change in the SPR. The rΔIm represents the ratio of incidence measures over 2 consecutive time periods, where a value of 2 represents a 2-fold increase and a value of 0.5 represents a 2-fold decrease in the incidence of malaria. It is important to note that the relationship between SPR and the incidence of malaria is neither linear nor proportional. For example, a 5% absolute decline in SPR from 50% to 45% corresponds to an 18% reduction in the incidence of malaria (rΔIm = 0.82) while the same 5% absolute decline in SPR from 10% to 5% corresponds to a 53% reduction in the incidence of malaria (rΔIm = 0.47). Similarly, a 50% relative decline in SPR from 50% to 25% corresponds to 67% reduction in the incidence of malaria (rΔIm = 0.33) while the same 50% relative decline in SPR from 10% to 5% corresponds to a 53% reduction in the incidence of malaria (rΔIm = 0.47). Understanding the mathematical relationship between changes in SPR and changes in malaria incidence has important implications for evaluating the impact of interventions using temporal changes in SPR.

Date from the cohort used in this study offered a valuable opportunity to test the relationship between changes in SPR and changes in the incidence of malaria within a population of African children. Using the assumptions described above, estimated values of rΔIm were calculated for two, six, and 12 month intervals and compared them to the corresponding observed values. While the SPR produced reasonable estimates of rΔIm over all three intervals, there was some bias in the estimated values due to the fact that the incidence of non-malaria fevers varied over time. Indeed, the incidence of non-malaria fevers showed marked seasonality as well as general decline over the course of the study. These two variations had differing effects, with seasonal changes in the incidence of non-malaria fevers primarily affecting estimated changes in the incidence of malaria over short time intervals (two months), and an overall decline affecting longer time intervals (12 months). The six month interval produced the best predictions because it grouped the incidence of non-malaria fevers into relatively equal seasons while remaining short enough to minimize the effect of the general decline in the incidence of non-malaria fevers over time. Overall, these results show the significant and varied effects that changing non-malaria fever rates can have on the accuracy of SPR in predicting changes in the incidence of malaria.

These results also demonstrate a consistent seasonal variation in the incidence of malaria and a decline in the incidence of malaria as age increases, both of which are well documented in other areas of Africa [13]. Substantial changes in the incidence of malaria may be due to these or other factors rather than the direct effect of malaria control interventions. As efforts to reduce the burden of malaria intensify, it is prudent to consider secular trends in malaria incidence when interpreting the effects of malaria control interventions on any surveillance data, including SPR.

## Conclusion

The SPR has several advantages as a surrogate measure for changes in the incidence of malaria using routine surveillance data. Surveillance using SPR applies a consistent and accepted case definition of malaria, is easy to integrate into most health management information systems, and can be done at a fraction of the cost of measuring the true incidence of malaria. SPR can be a useful measure of the impact of control interventions with several important considerations. First, accurate measurement of SPR requires the availability and utilization of quality laboratory diagnostic testing. Second, a change in SPR does not correspond to a proportional or linear change in the incidence of malaria. Third, when fever is used as the criterion for laboratory testing, a change in the incidence of non-malaria fevers can result in a change in SPR that does not reflect the true change in the incidence of malaria. Forth, additional factors such as age and seasonality can affect the incidence of malaria and may exaggerate or conceal the impact of interventions. Lastly, while SPR can be used to estimate relative changes in the incidence of malaria over time, it cannot estimate the actual incidence of malaria in a target population. Additional studies are needed to better understand the use of SPR in the context of surveillance programmes. In this study, presumable all febrile episodes were captured, laboratory testing was performed on all patients who presented with a fever, and laboratory testing for malaria was highly accurate. In contrast, most malaria surveillance systems only obtain a subset of fevers in a target population and are often limited by incomplete and inaccurate laboratory testing. The effect of these factors on the ability of the SPR to accurately estimate changes in the incidence of malaria may have important implications for the interpretation of surveillance using SPR in real world settings.

## Competing interests

The authors declare that they have no competing interests.

## Authors' contributions

HB, DNM, MRK, PR and GD contributed to study design and oversight. TPJ, GD, and DF contributed to methodology, data analysis, interpretation of the results and drafting of the manuscript. All authors read and approved the final manuscript.
